# Methodological Shortcomings in a Study of Peripheral Circulatory Complications in Type 2 Diabetes

**DOI:** 10.1002/edm2.70230

**Published:** 2026-04-30

**Authors:** Ahmar Jan Qureshi, Muhammad Riyyan Tariq Tagga, Raghabendra Kumar Mahato

**Affiliations:** ^1^ CMH Multan Institute of Medical Sciences Multan Pakistan; ^2^ Gandaki Medical College Teaching Hospital and Research Center Pokhara Nepal

**Keywords:** diabetes mellitus‐type 2, mortality, peripheral vascular diseases

## Abstract

**Introduction:**

This critique examines the methodological quality of a recent study by Shahid et al. on mortality trends related to peripheral circulatory complications in patients with type 2 diabetes mellitus using the CDC WONDER database.

**Methods:**

We critically appraised the original study's methodology, including the consistency of reported mortality rates, age group data presentation, classification of urban and rural areas, reporting of race and ethnicity data, place of death analysis and causal inferences drawn from the ecological design.

**Results:**

Several methodological shortcomings were identified: inconsistent reporting of mortality rate denominators (per 100,000 vs. per 1,000,000 population); selective reporting of age group data in the main text; use of a non‐standard threshold (500,000 population) for classifying urban and rural areas; inconsistent use of the ‘Non‐Hispanic’ qualifier for race and ethnicity categories; percentages summing to over 100% in the place of death analysis; and unsupported causal claims in the discussion.

**Conclusions:**

While the original study addresses an important public health concern, the identified methodological issues limit the interpretability of its findings. Addressing these concerns through consistent denominators, complete age‐stratified reporting and standard classifications would strengthen future research in this area.

To the Editor,

We read with interest the article by Shahid et al. [1], which examines mortality trends related to peripheral circulatory complications in patients with type 2 diabetes mellitus using the CDC WONDER database. The authors address an important public health concern and highlight persistent disparities across demographic and geographic strata. However, several methodological issues warrant careful consideration and limit the interpretability of the findings.

The first concern relates to inconsistency in the reporting of mortality rates. In the Results section, the authors state that the average age adjusted mortality rate was 11.08 per 100,000 individuals. Later in the same section and in the accompanying figures, rates are presented per 1,000,000 population. This is not a minor typographical oversight; it changes the reported values by an order of magnitude. A rate of 11.08 per 100,000 is equivalent to 110.8 per 1,000,000. Without a uniform denominator, readers cannot ascertain the true burden of peripheral circulatory complication‐related mortality, and meaningful comparison with other studies becomes impossible. The authors must apply a consistent denominator throughout the text, tables and figures.

A second issue concerns the presentation of age group data. The Methods section specifies that age groups were divided into 10‐year intervals ranging from 15 years to 85 years and older. Although the supporting information provides a complete breakdown of crude mortality rates across all age groups, the main text only reports rates for the 45–54 years and 85 years and older categories. This selective reporting in the main text limits the reader's ability to readily assess mortality patterns across the full age spectrum. The authors should consider summarising the full age distribution in the main results or explicitly directing readers to the supplementary table where these data appear.

A third methodological concern relates to the classification of urban and rural areas. The authors define urban areas as having a population of 500,000 or more and rural areas as those below 500,000. This definition deviates from the widely used National Center for Health Statistics Urban–Rural Classification Scheme, which classifies non‐metropolitan areas as having a population below 50,000, medium and small metropolitan areas as 50,000–999,999, and large metropolitan areas as ≥ 1,000,000 [2]. By using a threshold of 500,000, many small metropolitan and micropolitan areas are reclassified as rural, potentially distorting true urban–rural differences. The authors should either justify their choice or adopt established standards to ensure comparability with existing literature.

A fourth issue pertains to the reporting of race and ethnicity data. In the Methods section, race and ethnicity are classified as Non‐Hispanic (NH) Asian or Pacific Islander, NH American Indian or Alaska Native, NH White and NH Black or African American. In the Results section and in Figure 5, however, the groups are referred to without the ‘NH’ qualifier. This inconsistency creates ambiguity about whether Hispanic individuals were included in these racial categories. The terminology should be applied consistently throughout the manuscript.

Additional concerns emerge from the place of death analysis. The authors report that medical facilities accounted for 31,819 deaths (55.7%), nursing homes for 24,991 deaths (43.8%), home settings for 19,523 deaths (34.2%), hospices for 2508 deaths (4.4%) and other locations for 2493 deaths (4.4%). These percentages sum to well over 100%, indicating either overlapping categories or miscalculated denominators. The authors should present mutually exclusive data or clarify how the percentages were derived.

Finally, the discussion makes causal claims that are not supported by the ecological design. Statements attributing mortality patterns to rural hospital closures, opioid‐associated wound infections and the Special Diabetes Program for Indians are speculative, as the study did not include data on these factors. Such causal assertions should be reframed as hypotheses for future research.

Despite these methodological concerns, the study addresses a critically important topic and highlights troubling trends in mortality. Addressing the issues outlined above would strengthen the validity of the findings and provide a clearer foundation for the policy recommendations offered. Future studies should adopt consistent rate denominators, complete age‐stratified reporting and standard geographic and racial classifications to facilitate accurate interpretation and comparison across the literature.

## Author Contributions

**Raghabendra Kumar Mahato:** writing – review and editing, validation, supervision, resources. **Muhammad Riyyan Tariq Tagga:** investigation, visualization, writing – original draft, supervision. **Ahmar Jan Qureshi:** conceptualization, writing – original draft, methodology, project administration, data curation.

## Ethics Statement

The authors have nothing to report.

## Consent

The authors have nothing to report.

## Conflicts of Interest

The authors declare no conflicts of interest.

## Data Availability

Data sharing is not applicable to this article as no new data were created or analysed in this critique. The original study by Shahid et al. analysed publicly available data from the CDC WONDER database.
